# Independent Circulation of *Leishmania major* and *Leishmania tropica* in Their Respective Sandfly Vectors for Transmission of Zoonotic and Chronic Cutaneous Leishmaniasis Co-Existing in a Mixed Focus of Central Tunisia

**DOI:** 10.3390/pathogens11080855

**Published:** 2022-07-29

**Authors:** Mohammed Abdo Saghir Abbas, Jihene Lachheb, Ifhem Chelbi, Dorra Louati, Khalil Dachraoui, Slimene Ben Miled, Elyes Zhioua

**Affiliations:** 1 Pasteur Institute of Tunis, Unit of Vector Ecology, Tunis 1002, Tunisia; m.abbas9900@gmail.com (M.A.S.A.); ifhemc2001@yahoo.fr (I.C.); khalil.dachraoui@yahoo.com (K.D.); 2 Pasteur Institute of Tunis, Laboratory of Veterinary Microbiology, Tunis 1002, Tunisia; lachheb.jih@gmail.com; 3 Pasteur Institute of Tunis, Laboratory of Bioinformatics, Mathematic, and Biostatistics, Tunis 1002, Tunisia; dorra.louati@gmail.com (D.L.); slimane.benmiled@gmail.com (S.B.M.); 4 Mediterranean Institute of Technology, South Mediterranean University, Les Berges du Lac 2, Tunis 1053, Tunisia

**Keywords:** cocirculation, *Leishmania major*, *Leishmania tropica*, *Phlebotomus papatasi*, *Phlebotomus sergenti*, zoonotic cutaneous leishmaniasis, chronic cutaneous leishmaniasis, ecotones

## Abstract

Zoonotic cutaneous leishmaniasis (ZCL) and chronic cutaneous leishmaniasis (CCL) are known to overlap in Central Tunisia. Sandflies were collected using sticky traps and CDC light-traps set in rodent burrows at the ecotones surrounding the village, in houses, and in animal shelters during July–October 2017, 2018, and 2019. A total of 17,175 sandflies were collected during the three sandfly seasons and identified morphologically to species level. Of a total of 18 sandfly species reported in Tunisia, 16 were identified in this mixed focus of ZCL and CCL. Except for the rocky mountainous areas, *Phlebotomus papatasi* was the most abundant sandfly species in all biotopes. In the mountainous areas, *Phlebotomus sergenti* is the most abundant sandfly species belonging to the genus *Phlebotomus*. Female sandflies were tested for the presence of *Leishmania* species by PCR. The overall infection prevalence of sandflies with *Leishmania major* and *Leishmania tropica* was 0.42% and 0.065%, respectively. The sequencing of PCR-amplified ITS1 products showed that *L. major* is the predominant species in all biotopes and transmitted mostly by *P. papaptasi* followed by *Phlebotomus longicuspis* and *Sergentomyia* species. *Leishmania tropica* was detected in *Phlebotomus sergenti* and in *Phlebotomus longicuspis* collected in bedrooms and in the ecotone of rocky mountainous areas. Our results provided strong evidence that the proximity of human settlements to biotopes of rodent reservoir hosts of *L. major* and of *L. tropica* resulted into the cocirculation of both *Leishmania* species leading to a mixed focus of ZCL and CCL. The epidemiology of leishmaniases in North Africa is highly complex by the high diversity of sandfly vectors and their associated *Leishmania* species, leading to a mixed form of cutaneous leishmaniasis. It is of major epidemiological importance to point to the risk of spillover from rural to urban areas leading to the anthroponization of cutaneous leishmaniasis. Therefore, efficient control to reduce the indoor abundance of sandfly vectors in order to reduce the incidence of leishmaniases is urgently needed.

## 1. Introduction

Leishmaniases are vector-borne diseases caused by *Leishmania* protozoan parasites and they are transmitted to humans by the bite of infected female sandflies. Leishmaniases are widespread across 98 countries and 3 territories on 5 continents, with more than 58,000 visceral leishmaniasis cases (VL) and 220,000 cutaneous leishmaniasis cases (CL) per year [1]. The two forms of leishmaniasis coexist in Tunisia with a higher prevalence of CL compared to VL [1]. In Tunisia, CL is caused by three different *Leishmania* species: *Leishmania major*, *Leishmania infantum*, and *Leishmania tropica* (synonym, *Killicki*), etiologic agent of zoonotic cutaneous leishmaniasis (ZCL), sporadic cutaneous leishmaniasis (SCL), and chronic cutaneous leishmaniasis (CCL), respectively [2,3,4].

Sporadic cutaneous leishmaniasis caused by *L. infantum* is distributed in the humid, subhumid and semiarid biogeographical areas of Northern Tunisia and occurs sporadically with 50–150 cases per year [5]. To date, the life cycle of SCL has not yet been elucidated. Zoonotic cutaneous leishmaniasis (ZCL) caused by *L. major* is transmitted by the sandfly vector *Phlebotomus papatasi* [6,7]. *Psammomys obesus*, *Meriones shawi*, and *Meriones libycus* are the principal reservoir hosts of *L. major* [8,9,10,11,12]. *Mustela nivalis*, *Paraechinus aethiopicus*, *Atelerix algirus*, *Ctenodactylus gundi*, and *Psammomys vexillaris* are considered as potential reservoirs for *L. major* [13,14,15,16]. Annually, thousands of ZCL cases are reported from Central and Southern Tunisia [17,18], with the governorate of Sidi Bouzid alone having an estimated annual ZCL incidence rate of 669.7 per 100,000 inhabitants [19]. The population estimated to be at risk in the governorates of Kairouan, Sidi Bouzid, and Gafsa, the most endemic for ZCL, represents 87% of the total population at risk [20]. Chronic cutaneous leishmaniasis (CCL) occurs in hypoendemic microfoci located in the arid, rocky, mountainous areas of Southeastern Tunisia [21,22]. CCL is less prevalent than ZCL with 50–150 cases per year [5]. In Southeastern Tunisia, CCL is caused by *L. tropica* and transmitted by *Phlebotomus sergenti* [23]. The North African gundi (*Ctenodactylus gundi*) is considered as a putative reservoir host of *L. tropica* [24]. CCL is spreading towards the center and the southwest of Tunisia, leading to mixed foci in predominantly endemic ZCL areas [2,25,26,27]. The overlap of human cutaneous leishmaniasis due to *L. major* and to *L. tropica* is an increasingly important public health problem, as reported in the southeast [22], in the southwest [25], and in the center [25,26,27]. In the present study, an entomological investigation was carried out aiming to identify sandfly species and circulating *Leishmania* species in a mixed focus of ZCL with sporadic cases of CCL in Central Tunisia.

## 2. Material and Methods

### 2.1. Study Site

**Figure 1:**
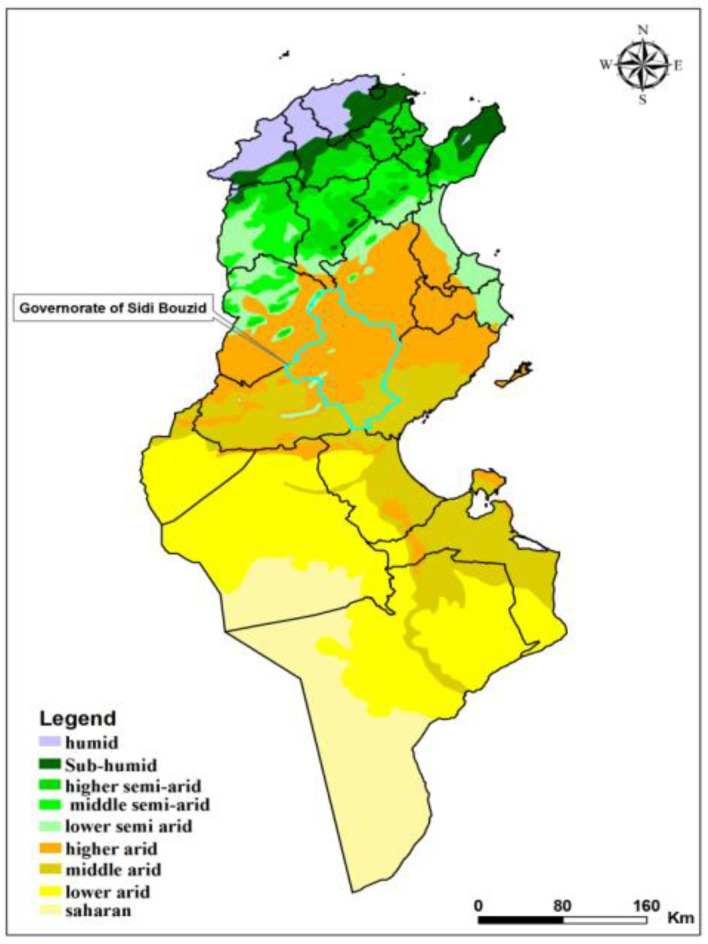
Bioclimatic map of Tunisia showing in the governorate of Sidi Bouzid.

The study was carried out in the governorate of Sidi Bouzid situated in an arid bioclimatic zone [28] (Figure 1), a highly endemic area with multiple foci of ZCL located in Central Tunisia [19]. The average annual incidence rate of ZCL was 666.7/100,000 inhabitants in the governorate of Sidi Bouzid and the dynamics of ZCL incidence are significantly heterogenic, occurring in outbreaks and clustering in space and time [19].

**Figure 2:**
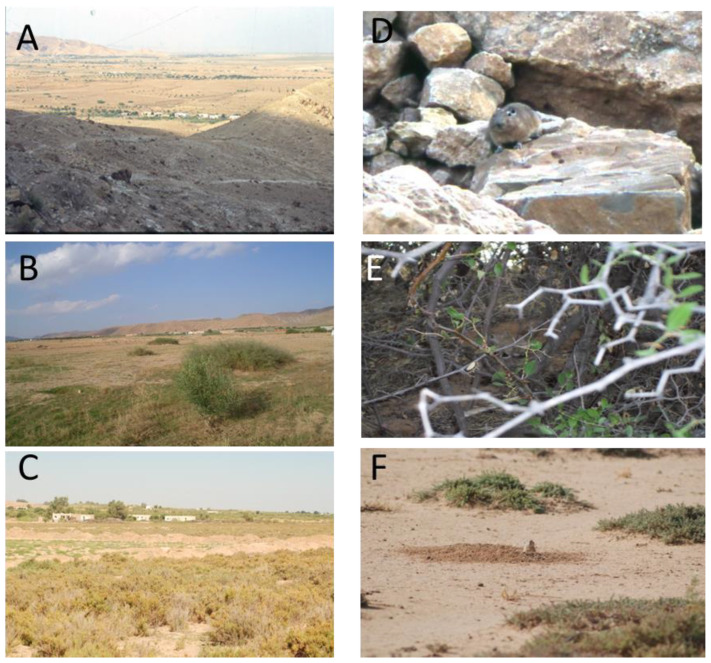
Biotopes of a mixed focus of zoonotic cutaneous leishmaniasis and chronic cutaneous leishmaniasis. The village is situated at the flank of the rocky mountainous areas which are the natural habitat of *Ctenodactylus gundi* (Figure 2**A**,**D**), and it is surrounded by agricultural fields harboring jujube trees which are the natural habitat of *Meriones shawi* (Figure 2**B**,**E**), and by nonagricultural fields made of chenopods, which are the natural habitat of *Psammomys obesus* (Figure 2**C**,**F**).

The study was performed in the village of Gouleb (9° 36′ E, 34° 48′ N), located in the delegation of Souk Jedid, within the governorate of Sidi Bouzid, a highly endemic focus of ZCL with an annual incidence of 3822.8 cases per 100,000 inhabitants [29], and it is considered as an emerging focus of CCL [30]. A hypoendemic focus of CCL has been reported in the delegation of Meknassy, which is adjacent to the delegation of Souk Jedid where the village of Gouleb is located [4,26]. The village is situated at the flank of the rocky mountainous areas which is the natural habitat of *C. gundi* [15] (Figure 2A,D). On the other side, the village is surrounded by agricultural fields harboring jujube trees (Figure 2B,E) and by nonagricultural fields made of chenopods, which are the natural habitat of *M. shawi* and *P. obesus* (Figure 2C,F), respectively. Thus, in the study sites, three ecotones which are the interface between human settlements and natural ecosystems were considered [31].

### 2.2. Sandfly Trapping and Identification

Our entomological investigation was carried out in the village of Gouleb during three consecutive years (2017–2019). In Tunisia, the phenology of *P. papatasi* is characterized by two main peaks of activity: one in June–July and a second, larger peak in September–October [18]. Each season, sandflies were collected during July–October by using CDC light traps (LT) and by sticky traps (ST) placed inside houses, animal shelters located in peridomestic areas, between rocks in the mountainous areas, in burrows of *M. shawi* and of *P. obesus*. Traps were placed from dusk to dawn and brought back the following morning to the laboratory. The collected sandflies were dissected to remove the head and the genitalia for species identification and the rest of the body was placed in a 1.5 mL microfuge tube to be examined for the presence of *Leishmania* DNA. Sandflies were identified at the species level by using the identification keys of Croset et al. [32] with special attention given to the atypical form of female *P. perniciosus* that could be confused with *P. longicuspis* [33,34]. Following identification, unfed female sandflies were pooled with up to a maximum of 30 specimens per pool based on the date of collection, species, and biotype and then stored in phosphate-buffered saline (PBS) for molecular analysis.

### 2.3. Detection of Leishmania DNA in Female Sandflies

Because this study deals with the circulation of two forms of cutaneous leishmaniasis in one focus, we used two different methods to detect *Leishmania* infection. Firstly, we used a nested-PCR-based schizodeme method targeting the partially conserved region of the kinetoplast minicircle DNA, enabling *Leishmania* species discrimination on the basis of PCR amplicon size, where *L. tropica* generated a 750 bp product, *L. infantum* produced a 680 bp product, whereas the product size of *L. major* was 560 bps [35]. Secondly, we used a nested PCR of a partial region of ITS-rDNA gene allowing the sequencing of detected *Leishmania* DNA as described by Parvizi et al. [36,37,38].

Female sandflies in pools were homogenized in 200 μL of PBS through high-speed shaking using the automated Tissue Lyser LT (Qiagen, Hilden, Germany) with glass beads. The mixture was clarified by centrifugation at 6000× *g* for 2 min for use in DNA extraction with a Qiagen DNA Mini Kit (Qiagen, Hilden, Germany) according to the manufacturer’s instructions. Extracted DNA from female sandflies was screened for infections with *Leishmania* species by a nested PCR based on the schizodeme method targeting the partial conserved region of the kinetoplast minicircle DNA as previously described [35]. This method allows the discrimination of *Leishmania* species originating from North Africa based on the PCR amplicon size. The nested PCR product sizes for *L. tropica*, *L. major* and *L. infantum* were 750, 560 bp and 680 bp, respectively [35]. The first PCR was performed using the Taq DNA recombinant polymerase kit (Invitrogen, Waltham, MA, USA) in 25 μL containing: 2.5 μL 10X buffer, 1.5 μL MgCl2 (50 mM), 1 μL dNTP mix (10 mM), 1 μL of each reverse and forward primers CSB2xF/CSB1xR (10 μM), 0.25 μL Taq DNA polymerase (5 U/μL), 12.75 μL nuclease-free water and 5 μL of extracted DNA. The nested PCR was carried out in 50 μL containing 3 μL of the first PCR step DNA product and 47 μL of a mixture containing: 5 μL 10X buffer, 3 μL MgCl2 (50 mM), 2 μL dNTP mix (10 mM), 2 μL of each reverse and forward internal primers 13Z/LIR (10 μM), 0.5 μL of Taq DNA polymerase (5 U/μL) (Invitrogen, Waltham, MA, USA), and 32.5 μL of RNase and DNase-free H_2_O. Optimized cycling conditions for the first and second PCR steps were performed as follows: 94 °C for 5 min followed by 35 cycles, repeating denaturation at 94 °C for 30 s, annealing at 55 °C for 60 s and elongation at 72 °C for 90 s, and an extension step at 72 °C for 10 min. Previously extracted *L. tropica* (MHOM/TN/88/TAT3) and *L. major* (MHOM/TN/97/LPN162) DNA were used as a positive control for *Leishmania* detection. Cross-contamination was monitored by negative controls for sample extraction and PCR assay. Amplification products of the nested PCR were then visualized by electrophoresis in 1.5% agarose gel supplemented with ethidium bromide under UV-light transillumination. Positive PCR product sizes were estimated according to 100 bp molecular weight (Invitrogen, Waltham, MA, USA) to identify sandfly-associated *Leishmania* species.

In this study, the infection of sandfly species by *Leishmania* species is reported using the minimum infection rate (MIR) which is calculated by: ([number of positive pools/total number of tested sandflies] × 100) [39].

### 2.4. Detection of Leishmania DNA, DNA Sequencing and Phylogenetic Analysis

Extracted DNA was screened for infections of *Leishmania* species by a nested PCR of a partial region of the ITS-rDNA gene as previously described [36,37]. The first amplification steps were performed using the Taq DNA recombinant polymerase kit (Invitrogen, Waltham, MA, USA) in 50 μL reaction containing: 5 μL 10X buffer, 3 μL MgCl_2_ (50 mM), 2 μL dNTP mix (10 mM), 1 μL of each reverse and forward primers IR1/IR2 (10 μM), 0.5 μL Taq DNA polymerase enzyme and 10 μL of total extracted DNA. The nested PCR was carried out in 50 μL containing 2 μL of the first PCR step DNA product and 48 μL of mixture containing: 5 μL 10X buffer, 3 μL MgCl_2_ (50 mM), 2 μL dNTP mix (10 mM), 1 μL of each reverse and forward internal primers ITS1F/ITS2R4 (10 μM) and 0.5 μL of Taq DNA polymerase (Invitrogen, Waltham, MA, USA). Optimized cycling conditions for the first and second PCR step were performed as follows: (i) 94 °C for 3 min followed by 40 cycles of 94 °C for 60 s, 58 °C for 60 s and 72 °C for 90 s, followed by a final extension step (72 °C) for 10 min; (ii) nested PCR with 94 °C for 3 min followed by 5 cycles of 94 °C for 60 s, 55 °C for 60 s and 72 °C for 60 s, and 35 cycles of incubation at 94 °C, 59 °C and 72 °C for 60 s each. The extension step was continued for 10 min at 72 °C. Cross-contamination was monitored by negative controls for sample extraction and PCR solutions for the PCR test. Amplification products of the nested PCR were separated in 2% agarose gel stained with ethidium bromide and visualized under UV-light illumination. Positive PCR products were directly sequenced to identify sandfly-associated *Leishmania* species.

The 462bp nested PCR products were purified by the ExoSAP-IT method using the Exonuclease-I and the Shrimp Alkaline Phosphatase were sequenced in both directions using a Big Dye Terminator ready reaction cycle sequencing v3.1 kit (Applied Biosystems, Waltham, MA, USA) with forward and reverse nested PCR primers (ITS1F/ITS2R4) [38]. The resulting consensus sequences were deduced by aligning the respective forward and reverse sequences using CLUSTAL_W 1.4 implemented in MEGA v.5.22 [40]. In addition to the studied sequences, several *Leishmania* species sequences, including 1 *L. brazeliensis*, 13 *L. tropica*, and 33 *L. major*, were selected from the GenBank database. Phylogenetic analysis was performed using the maximum likelihood analysis method and the Tamura-3 parameter model. The tree topology was supported by 1000 bootstrap replicates.

### 2.5. Data Analysis

#### Sandfly Species Diversity

Biological diversity was quantified by measuring richness and evenness for a better understanding of community structure [41]. To assess the sandfly fauna structure, the following ecological parameters and diversity indexes were calculated:

Relative abundance (ni): Number of sandfly species/Total number of sandflies in the sample.

Specific richness (S): number of species in the sample [41].

Specific diversity was also measured by the Shannon–Wiener index (*H*′) that takes into consideration the probability of encountering a specific species in a stand. To better discuss this Shannon index, it is often accompanied by the Piélou equitability index (J), or equirepartition index (E). Its formula corresponds to the ratio between *H*′ and Hmax: E = *H′*/Hmax. This index varies between 0 and 1. If it tends towards E = 1, then the species present in the stand have identical abundances. If it tends towards E = 0, then we are in the presence of an imbalance where a single species dominates the entire stand.

$$H^{\prime }\;=\;-\overset{S}{\underset{i=1\;}{\sum }}pi\;log\left(pi\right)$$

*pi* = the proportional abundance or percent abundance of a species present (*pi* = ni/N).
ni = the number of individuals counted for the species.
N = the total number of individuals counted, all species combined.
*S* = the total or cardinal number of the list of present species.

Statistical analysis was performed using python Jupiter notebook. Fisher’s exact test was applied to a 2 × 2 contingency table to compare the relative abundance of sandfly species in different habitats and their distribution within infected sandflies. The significance level was set at 5%.

## 3. Results

### 3.1. Sandfly Fauna

In the focus on Gouleb, 16 sandfly species were identified, belonging to two genera, *Phlebotomus* (ten species) and *Sergentomyia* (six species). Among sandfly species of the genus *Phlebotomus*, *Phlebotomus (Phlebotomus) papatasi* was the most abundant species followed by *P. (Larroussius) longicuspis* and *P. (Paraphlebotomus) sergenti*. The remaining sandfly species of the genus *Phlebotomus* were less prevalent including: *P. (Larroussius) perniciosus*, *P. (Larroussius) perfiliewi*, *P. (Larroussius) ariasi*, *P. (Larroussius) langeroni*, *P. (Paraphlebotomus) alexandri*, *P. (Paraphlebotomus) riouxi*, and *P. (Paraphlebotomus) chabaudi*. Among sandfly species of the genus *Sergentomyia*, *Sergentomyia (Sergentomyia) fallax* was the most prevalent followed by *S. (Sergentomyia) minuta*. The remaining sandfly species of the genus *Sergentomyia* were less prevalent including: *S. (Sergentomyia) antennata*, *S. (Grassomyia) dreyfussi*, *S. (Sintonius) christophersi*, and *S. (Sintonius) clydei* (Table 1).

Of the total collected sandflies in all biotopes during the three sandfly seasons, *P. (Phlebotomus) papatasi*, *S. (Sergentomyia) fallax*, *S. (Sergentomyia) minuta* and *P. (Larroussius) longicuspis* corresponded to 93% of all specimens. The 12 remaining species were less prevalent and represented 7% of the total sandfly fauna. Since the Piélou’s equitability index E = *H′*/Hmax was 0.55, the sandfly fauna was imbalanced with four dominant species in the entire stand.

The relative abundance of predominant sandfly species varied significantly among biotopes (Table 2). Among sandfly species of the genus *Phlebotomus*, *P. sergenti* was the predominant sandfly in the mountainous areas, the natural habitat of the gundi, compared to other biotopes (*p* < 0.05). *Phlebotomus papatasi* was less prevalent in gundi’s biotope, predominant in bedrooms, animal shelters located in the peridomestic areas, and rabbit holes, and highly abundant in burrows of *M. shawi* and *P. obesus* (*p* < 0.05). *Phlebotomus longicuspis* was abundant in animal shelters located in the peridomestic areas, bedrooms, and to a lesser extent, in gundi’s biotope. *Sergentomyia fallax* and *S. minuta* had the same patterns of distribution, being the most abundant in gundi’s biotope followed by bedrooms, and animal shelters located in the peridomestic areas (Table 2).

### 3.2. Leishmania Detection

A total of 957 pools of unfed female sandflies were screened for *Leishmania* infection by the nested-PCR-based schizodeme method targeting the partially conserved region of the kinetoplast minicircle DNA. Thirty pools were found to be positive for *Leishmania* DNA. Hence, the overall minimum infection rate of sandflies with *Leishmania* DNA was 0.5% (30/6187). Sandflies forming positive pools were collected from bedrooms, animal shelters located in the peridomestic areas, gundi’s biotope, and rodents’ burrows (Table 3).

The overall infection prevalence of sandflies with *L. major* and *L. tropica* was 0.42% (26/6187), and 0.065% (4/6187), respectively. Among the 30 positive pools, 26 were positive for *L. major* DNA (86.6%), and 4 positive for *L. tropica* DNA (13.3%) (Table 3). Of a total of 26 positive pools for *L. major* DNA, 16 (61.5%) were detected in pools of *P. papatasi*, 4 in pools of *S. fallax* (15.4%), 3 in pools of *S. antennata* (11.5%), 1 in a pool of *S. minuta* (3.8%), 1 in a pool of *P. longicuspis* (3.8%), and 1 in a pool of *P. sergenti* (3.8%) (Table 3). Among positive pools for *L. tropica* DNA, two were detected in two pools of *P. sergenti* and two were detected in two pools of *P. longicuspis* (Table 3).

**Figure 3:**
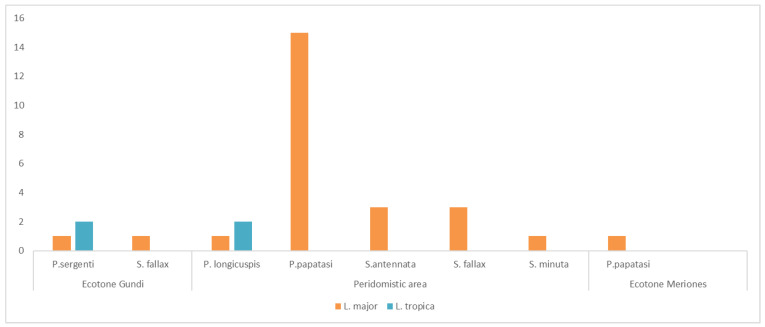
Infected sandflies according to *Leishmania* species, sandfly species, and biotopes.

All positive pools of sandflies collected from bedrooms (N = 7) were infected only with *L. major* (five pools of *P. papatasi* (71.4%), one pool of *S. fallax* (14.2%), and one pool of *S. antennata* (14.2%)) (Figure 3). Among the 18 positive pools of sandflies collected from the peridomestic areas including rabbits’ holes and animal shelters, 10 (55.5%) were *L. major-*DNA-infected pools of *P. papatasi*, 5 *L. major-*DNA-infected pools of *Sergentomyia* sp. (27.7%), 1 *L. major-*DNA-infected pool of *P. longicuspis* (5.5%), and 2 *L. tropica-*DNA-infected pools of *P. longicuspis* (11.1%) (Figure 3). One pool of *P. papatasi* collected from burrows of *M. shawi* and/or *P. obesus* was positive for *L. major* DNA (Figure 3). Despite the fact that *L. major* was detected in *Sergentomyia* species, *P. sergenti*, and *P. longicuspis*, it remained highly associated with *P. papatasi* (*p* < 0.05). In the rocky mountainous areas, which are the natural biotope of *C. gundi*, in addition to two pools of *P. sergenti* positive for *L. tropica* DNA (50%), two pools (one *S. fallax* and one *P. sergenti*) were positive for *L. major* DNA. Statistically, an association was shown between *P. sergenti* and *P. longicuspis* towards *L. tropica* (*p* < 0.05).

### 3.3. Leishmania DNA Sequencing and Phylogenetic Analysis

From the 30 PCR-positive products for *Leishmania* DNA which were sequenced, only 18 were readable. The alignment of ITS sequences obtained confirmed that samples corresponded to 3 *L. tropica* and 15 *L. major*. Three sequences corresponding to three *L. tropica* (from two *P. sergenti* and one *P. longicuspis*) were selected and deposited in GenBank under accession numbers OK338429, OK354361, and ON243921. Fifteen sequences corresponding to *L. major* (12 from *P. papatasi*, 1 from *P. longicuspis*, 1 from *P. sergenti*, and 1 from *S. fallax*) were deposited in GenBank under accession numbers OK355181, OK357907, OK374713, ON243631, ON243638, ON243641, ON243845, ON243847, ON243867, ON243871, ON243877, ON243878, ON243881, ON243882, and ON243887.

A phylogenetic analysis was performed to observe the phylogenetic relationships of ITS among species. The identified *Leishmania* DNA sequences are closely related to the reference sequence of *L. major* and *L. tropica* with (96.8–99%) and (99–100%) of identity, respectively.

**Figure 4:**
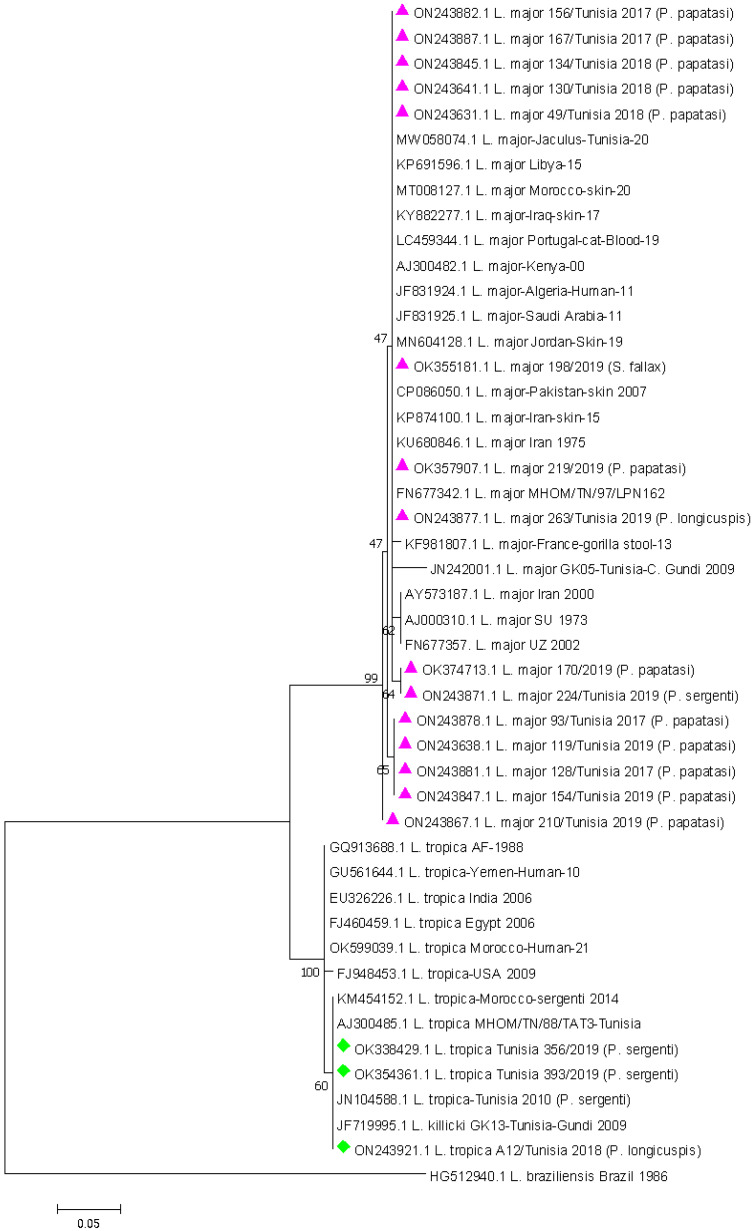
Phylogenetic tree based on partial *Leishmania* ITS-rDNA 5.8 s sequences.

The sequence analyses showed that Tunisian *L. major* sequences found in this study (OK355181, OK357907, OK374713, ON243631, ON243638, ON243641, ON243845, ON243847, ON243867, ON243871, ON243877, ON243878, ON243881, ON243882 and ON243887) were closely related to a Tunisian sequence (MHOM/TN/97/LPN162 accession number FN677342) isolated in 1997 with (98 to 100%) identity at the nucleotide level. Regarding sequences of *L. tropica* (OK338429, OK354361 and ON243921), they showed 100% identity to Tunisian sequences (MHOM/TN/88//TAT3 accession number AJ300485) isolated in 1988. Phylogenetic reconstructions revealed clustering of obtained *Leishmania* sequences within *L. major* and *L. tropica* genetic clades. The phylogenetic branch was supported by a high bootstrap value of 99% and 100% for *L. major* and *L. tropica*, respectively (Figure 4).

The evolutionary history was inferred by using the maximum likelihood method based on the Tamura three-parameter model. The 1000 bootstrap pseudo-replication values were reported at nodes. The scale bar represents 0.05% divergence with branch lengths measured in the number of substitutions per site. The analysis involved 47 nucleotide sequences aligned using the CLUSTAL algorithm. All positions containing gaps and missing data were eliminated. There was a total of 155 positions in the final dataset. Evolutionary analysis was conducted in MEGA7. The sequences obtained are marked with a pink triangle and a green diamond. *Leishmania braziliensis* was used as the outgroup.

## 4. Discussion

Despite the high annual prevalence of ZCL in the delegation of Souk Jedid where the village of Gouleb is located [18,19,20,29], little is known regarding the epidemiological aspects of the coexistence of ZCL and CCL in this area. Species identification of the sandfly vectors and etiological-agent typing in these vectors represent steps forward in understanding the epidemiology of leishmaniasis, which should lead to the implementation of improved disease control programs. Taking into account the coexistence of ZCL and CCL, in a nearby focus of CCL [26], the focus of Gouleb is of particular epidemiological importance. The present study aimed: (1) to clarify the diversity and the abundance of sandflies, and (2) to identify the *Leishmania* species infecting sandfly species in this emerging CCL within a focus of a predominantly ZCL.

Following this biological approach, we quantified the species diversity of sandfly populations in and around houses of CL cases due to *L. major* and in biotopes of the reservoir host *Meriones*. Species diversity was low in all habitats. As previously reported in ZCL foci, the proven vector of *L. major* in Tunisia, *P. papatasi*, was the dominant species [31]. Other species were probably insufficiently abundant in the *Meriones* habitat to be easily captured in and around the houses. In these recently established settlements, *P. papatasi* is probably the first species, or the only species, to invade new houses, because of its specific behavior.

Of a total of 18 sandfly species reported from Tunisia [42,43], 16 species were collected from the site of Gouleb, representing 88.8% of the sandfly fauna reported from Tunisia. Except in the mountainous areas, *P. papatasi* was the predominant sandfly species in all biotopes including bedrooms, peridomestic areas, and agricultural fields surrounding the village harboring burrows of rodents which are reservoir hosts of *L. major*.

In addition, *P. papatasi* was the most prevalent *L. major*-infected sandfly species in all biotopes including rodents’ burrows. Recently, it was shown that ZCL incidence is significantly higher in the ecotones of *M. shawi* compared to ecotones of *P. obesus* [31]. This finding could be explained by the high infection prevalence of *M. shawi* with *L. major* reaching 53% in autumn compared to the infection prevalence of *P. obesus* (41%) [11], and by its migratory behavior leading to the dispersal of ZCL [44]. Considering that the flight range of *P. papatasi* is around 0.75 km [45], increases in densities of *L. major-*infected *P. papatasi* in the ecotone of *M. shawi* expand the overlap of the infected ZCL vector with human habitations and communities contributing to the emergence of epidemics among naïve human populations [46]. Taking into account that *P. papatasi* (i) is highly associated with burrows of *M. shawi* at the ecotone level [31] and (ii) is the most abundant sandfly species indoors and is highly endophilic with a trophic preference for humans and rodents [46], consequently, ZCL is the predominant form of CL in the focus of Gouleb [19,29].

*Phlebotomus sergenti* is the predominant sandfly species belonging to the genus *Phlebotomus* in the mountainous areas of the village of Gouleb, a natural biotope of the gundi [15]. Indoors, *P. sergenti* is endophilic but it may take longer for this sandfly species to invade houses compared to the most abundant species *P. papaptasi*, as has been reported by Jaouadi et al. [47] in the same area and by Tabbabi et al. [23] in a mixed focus of ZCL and CCL in Southeastern Tunisia. Previous studies reported the detection of *L. tropica* in *P. sergenti* collected from bedrooms [47,48]. In the present work, we reported for the first time the detection of *L. tropica* in *P. sergenti* collected from the rocky mountainous areas of the village, a natural biotope of the gundi. In addition, *L. tropica* was detected in gundi [49], and also in *P. sergenti* trapped from a bedroom in a site near to the village of Gouleb [47], and isolated from a human in the same region [27]. The aforementioned findings provide strong evidence to incriminate *P. sergenti* and *C. gundi* as the vector and the reservoir of *L. tropica*, respectively, in Central Tunisia, as has been reported from the Southeast of Tunisia [24,48].

In the present study, we showed that *P. sergenti* is totally absent from burrows of *M. shawi* and *P. obesus*; therefore, this sandfly species is not cavernicolous compared to *P. papatasi*. Thus, it is expected that *P. sergenti* will not be involved in the transmission of *L. major* between *M. shawi* and/or *P. obesus*. However, we reported for the first time the natural infection of unfed female *P. sergenti* collected from the rocky mountainous areas with *L. major*. Concomitantly, a high infection prevalence of gundi with *L. major* (30%, N = 23) trapped in a nearby site named Khabina was reported [15]. The detection of *L. major* DNA in *P. sergenti* does not mean necessarily that this sandfly species is permissive for *L. major*. *Phlebotomus sergenti* was described as specific vector only for *L. tropica* and not for *L. major* [50,51]. Kamhawi et al. [50] highlighted the role of sandfly midgut lipophosphoglycan (LPG) receptors in *Leishmania* attachment and the impact of LPG on the vectorial competence of *P. sergenti* for only *L. tropica* and, consequently, this sandfly species is not permissive for other *Leishmania* species such as *L. major* and/or *L. donovani*. Since *P. sergenti* may not have the genetic characteristics of a single species [52], it is of major epidemiological importance to study the vectorial competence and the vectorial capacity of the North Africa strain of *P. sergenti* in the transmission of *L. major*.

In Central Tunisia, ZCL is the predominant form of CL caused by *L. major* due to the predominance of the sandfly vector *P. papatasi* in the peridomestic areas and its high infection prevalence with the parasite. However, the occurrence of sporadic human cases of CCL in Central Tunisia located at the flank of rocky, arid, mountainous areas is most probably due to the involvement of *P. sergenti* and the gundi in the transmission of *L. tropica*, and, subsequently, leading to the coexistence of ZCL and CCL. In Morocco, several studies conducted in historical foci of CL, due to *L. major* being close to mountainous areas where *P. sergenti* is the most commonly collected sandfly species, have shown the emergence of *L. tropica* in these foci [53,54,55]. The North African form of *L. tropica* tends to be zoonotic and endemic in rural areas compared to the Middle East form which is anthroponotic and endemic in urban cities [56]. Recently, several epidemics with hundreds of cases of *L. tropica* CL have been reported in several Moroccan cities [57,58]. It is of major epidemiological importance to point to the risk of spillover of CCL from rural settlements to urban areas in Tunisia [59], added to the risk of anthroponization which may lead to major outbreaks of cutaneous leishmaniasis due to *L. tropica* in major cities located in Central and Southern Tunisia. Further studies are needed to assess the risk of urbanization of CCL in Central Tunisia.

Among sandfly species belonging to the genus *Phlebotomus*, *P. longicuspis* is the second most dominant species in bedrooms and in animal shelters located in the peridomestic areas and are rare in the rocky mountainous areas as well as in burrows of *M. shawi* and *P. obesus*. The infection of *P. longicuspis* collected from animal shelters with *L. tropica* point to the potential role of this sandfly species in the transmission of CCL in Central Tunisia. Similar results were reported by Remadi et al. [60] from arid Central Tunisia. *Phlebotomus longicuspis* is abundant in the Saharan and arid bioclimatic zones with a relative abundance of 60% and 40%, respectively [61]. Recently, we showed the involvement of *P. longicuspis* in the transmission of *L. infantum* in highly irrigated areas of Central Tunisia [39]. Similar results were reported by Remadi et al. [60], and it is suspected to be the main vector of *L. infantum* in Southern Tunisia [61]. In Morocco, *P. longicuspis* is considered a vector of *L. infantum* in a CL focus where *P. sergenti* is confirmed as the main vector of *L. tropica* [62,63]. In Northern Algeria, *P. longicuspis* is suspected to be a competent vector of zoonotic visceral leishmaniasis in these areas [64]. As for *P. perniciosus*, *P. longicuspis* appears to be a permissive vector for *Leishmania* species.

Sandflies of the genus *Sergentomyia*, mainly *S. fallax*, *S. antennata*, and *S. minuta*, are predominant mainly in the rocky mountainous areas, indoors, animal shelters, and rodents’ burrows. Indoors, *L. major* was detected in *S. fallax* and *S. antennata*. In the rocky mountainous areas, *S. fallax* was shown to be infected with *L. major*. In animal shelters, *L. major* was detected in *S. fallax*, *S. antennata*, and *S. minuta*. It is important to point out that no *L. tropica* was detected in *S. fallax*, *S. antennata*, and *S. minuta* trapped in the rocky mountainous areas, the natural biotope of gundi. In an old emerging ZCL focus of Southern Tunisia (Gafsa), *L. major* was detected in *S. minuta* trapped in the peridomestic areas [65]. Here, we reported for the first time the detection of *L. major* in *S. fallax* and *S. antennata*. Our findings strongly suggest that *S. fallax*, *S. antennata*, and *S. minuta* are suspected vectors of *L. major* and the gundi is considered a potential reservoir host in the rocky mountainous areas close to communities, and may play a significant role in the transmission of ZCL.

In conclusion, the epidemiology of leishmaniases in North Africa is highly complex due to the high diversity of sandfly vectors and their associated *Leishmania* species, leading to a mixed form of CL caused by different pathogens, with the risk of spillover from rural to urban areas. Therefore, efficient control to reduce the indoor abundance of sandfly vectors to reduce the incidence of leishmaniases is urgently needed.

## Figures and Tables

**Table 1:** Sandfly fauna in the focus on Gouleb (2017–2019).

Species (Subgenus)	ST ♂/♀	Subtotal	LT ♂/♀	Subtotal	Total	(%)
*P. (Phlebotomus) papatasi*	1874/474	2348	3364/1897	5261	7609	(44.30)
*P. (Paraphlebotomus) sergenti*	68/19	87	70/89	159	246	(1.43)
*P. (Paraphlebotomus) alexandri*	15/8	23	67/44	111	134	(0.78)
*P. (Paraphlebotomus) chabaudi*	1/2	3	0/3	3	6	(0.03)
*P. (Paraphlebotomus) riouxi*	2/0	2			2	(0.01)
*P. (Larroussius) ariasi*	8/0	8	1/6	7	15	(0.09)
*P. (Larroussius) longicuspis*	29/0	29	1210/769	1979	2008	(11.69)
*P. (Larroussius) perfiliewi*	3/1	4	10/8	18	22	(0.13)
*P. (Larroussius) perniciosus*	40/2	42	14/17	31	73	(0.43)
*P. (Larroussius) langeroni*			0/2	2	2	(0.01)
*S. (Sergentomyia) fallax*	994/471	1465	1506/1195	2701	4166	(24.26)
*S. (Sergentomyia) minuta*	767/320	1087	765/376	1141	2228	(12.97)
*S. (Sergentomyia) antennata*	40/21	61	82/247	329	390	(2.27)
*S. (Grassomyia) dreyfussi*	2/1	3	53/191	244	247	(1.44)
*S. (Sintonius) christophersi*	1/1	2	2/19	21	23	(0.13)
*S. (Sintonius) clydei*	0/2	2	0/2	2	4	(0.02)
Total	3844/1322	5166	7144/4865	12,009	17,175	

**Table 2:** Sandflies caught by biotype (2017–2019).

	G.B	Bed	A.S	R.H	BMs	BPo	Total
Species	Su.T (%)	Su.T (%)	Su.T (%)	Su.T (%)	Su.T (%)	Su.T (%)	Total (%)
*P. papatasi*	63 (2.1)	1747 (33.13)	1623 (39.13)	1866 (88.31)	1799 (88.36)	511 (84.6)	7609 (44.3)
*P. sergenti*	100 (3.33)	77 (1.46)	55 (1.33)	14 (0.66)			246 (1.43)
*P. chabaudi*	5 (0.17)		1 (0.02)				6 (0.03)
*P. alexandri*	26 (0.87)	56 (1.06)	47 (1.13)	2 (0.99)	2 (0.1)	1 (0.17)	134 (0.78)
*P. riouxi*	2 (0.07)						2 (0.01)
*S. fallax*	1487 (49.55)	1895 (35.94)	502 (12.1)	105 (4.97)	118 (5.8)	59 (9.77)	4166 (24.26)
*S. minuta*	1145 (38.15)	659 (12.5)	277 (6.68)	39 (1.85)	90 (4.42)	18 (2.98)	2228 (12.97)
*S. antennata*	63 (2.10)	208 (3.94)	85 (2.05)	12 (0.57)	16 (0.79)	6 (0.99)	390 (2.27)
*S. dreyfussi*	24 (0.8)	115 (2.18)	103 (2.48)	3 (0.14)	1 (0.05)	1 (0.17)	247 (1.44)
*S. clydei*			1 (0.02)		2 (0.1)		4 (0.02)
*S. christophersi*	1 (0.03)	9 (0.17)	13 (0.31)		1 (0.05)		23 (0.13)
*P. longicuspis*	25 (0.83)	487 (9.24)	1416 (34.14)	67 (3.17)	6 (0.29)	7 (1.16)	2008 (11.69)
*P. perniciosus*	47 (1.57)	10 (0.19)	12 (0.29)	3 (0.14)	1 (0.05)		73 (0.43)
*P. perfiliewi*	3 (0.1)	10 (0.19)	7 (0.17)	1 (0.05)		1 (0.17)	22 (0.13)
*P. langeroni*			2 (0.05)				2 (0.01)
*P. ariasi*	10 (0.33)		4 (0.1)	1 (0.05)			15 (0.04)
Total	3001	5273	4148	2113	2036	604	17,175

Legend of abbreviations: G.B: gundi’s biotope, Bed: bedroom, A. S: animal shelter, R.H: rabbit hole, BMs: burrows of *Meriones shawi*, BPo: burrows of *Psammomys obesus*.

**Table 3:** *Leishmania*-infected sandflies according to biotypes.

Date	Biotype	Sandfly/Pool (Total)	Sandfly Species	*Leishmania* Species
13 July 2017	A. S	1 (435)	*P. longicuspis*	*L. tropica*
	Bed	4 (494)	*P. papatasi*	*L. major*
	A. S	24 (435)	*P. papatasi*	*L. major*
	Bed	3 (494)	*P. papatasi*	*L. major*
	A. S	1 (435)	*S. antennata*	*L. major*
5 September 2017	A. S	1 (136)	*P. papatasi*	*L. major*
	A. S	1 (136)	*S. antennata*	*L. major*
	R.H	19 (1062)	*P. papatasi*	*L. major*
19 September 2017	R.H	2 (277)	*P. papatasi*	*L. major*
	Bed	9 (154)	*S. fallax*	*L. major*
26 September 2017	Bed	3 (485)	*S. antennata*	*L. major*
	A. S	2 (215)	*S. fallax*	*L. major*
23 August 2018	A.S	1 (39)	*P. longicuspis*	*L. tropica*
7 September 2018	A. S	3 (82)	*P. papatasi*	*L. major*
12 September 2018	A. S	6 (260)	*S. fallax*	*L. major*
	R.H	8 (59)	*P. papatasi*	*L. major*
	Bed	2 (445)	*P. papatasi*	*L. major*
	A. S	1 (260)	*P. papatasi*	*L. major*
10 October 2018	A. S	1 (127)	*S. minuta*	*L. major*
25 September 2019	G.B	14 (210)	*S. fallax*	*L. major*
2 October 2019	R.B	6 (24)	*P. papatasi*	*L. major*
2 October 2019	A. S	3 (39)	*P. papatasi*	*L. major*
25 September 2019	Bed	2 (170)	*P. papatasi*	*L. major*
3 October 2019	A. S	5 (276)	*P. longicuspis*	*L. major*
25 September 2019	A. S	30 (279)	*P. papatasi*	*L. major*
24 September 2019	A. S	30 (695)	*P. papatasi*	*L. major*
24 September 2019	Bed	1 (219)	*P. papatasi*	*L. major*
17 October 2019	G.B	2 (389)	*P. sergenti*	*L. tropica*
23 October 2019	G.B	5 (181)	*P. sergenti*	*L. tropica*
2 October 2019	G.B	2 (224)	*P. sergenti*	*L. major*

Legend of abbreviations: A. S: animal shelter, Bed: bedroom, R.H: rabbit hole, G.B: gundi’s biotope, R.B: rodents’ burrows near houses.

## Data Availability

This study did not report any data.
